# P-1786. Implementation of a Clinical Pathway to Educate Clinicians on Changing Epidemiology

**DOI:** 10.1093/ofid/ofae631.1949

**Published:** 2025-01-29

**Authors:** Eric Roessler, Emily Landon, John Flores, R Marrs, Natasha N Pettit, Jennifer Pisano, Elizabeth Bell

**Affiliations:** University of Chicago, Chicago, Illinois; University of Chicago Medicine, Chicago, Illinois; University of Chicago Medicine, Chicago, Illinois; University of Chicago Medicine, Chicago, Illinois; University of Chicago Hospital, Chicago, Illinois; University of Chicago Medicine, Chicago, Illinois

## Abstract

**Background:**

Since 8/31/2022, 39,998 new arrivals have made Chicago their home, with the majority coming from Latin America. Epidemiological diversity in these countries of origin as well as infection risks encountered on the journey to the US have resulted in increased requests for advice from Infectious Diseases (ID) specialists and a need for rapid education of clinicians, raising the question of how to best address this knowledge gap.

Initial Triage Algorithm

**Figure:**
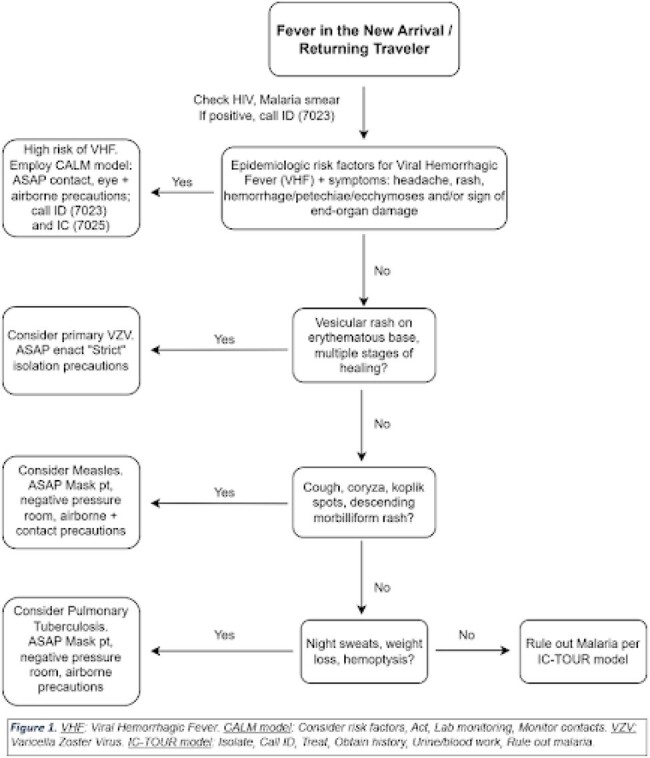

Clinicians are directed to this algorithm when first presented with a febrile new arrival or returning traveler.

**Methods:**

Using Chicago Department of Public Health (CDPH) data, Epic® (Verona, WI), CDC’s online information for clinicians, and expert advice, we quantified relevant epidemiologic changes, curated a list of high priority topics, and developed an algorithmic approach to fever in new arrivals for the general clinician. After three cycles of revision within our interdisciplinary Antimicrobial Stewardship Program (ASP), this document was uploaded to the highly utilized ASP website for all providers on 4/26/2024.

Differential Diagnosis Assistance after Malaria Ruled Out

**Figure:**
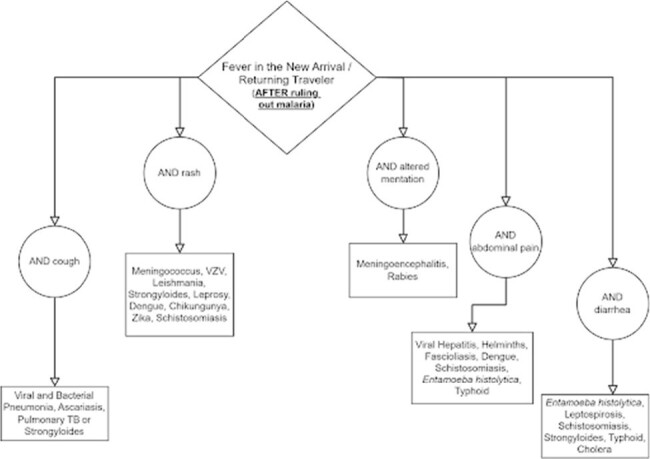

Clinicians are directed to this figure after completing the initial algorithm and ruling out malaria to consider additional neglected tropical diseases in their differential diagnosis.

**Results:**

On review of epidemiologic data, we found that Chicago had 0 Measles cases in 2019, 5 cases in 2023, and 64 cases in 2024 as of 4/23/2024. Varicella incidence in Chicago rose from a median of 53 cases/year during 2005-2022 to roughly 400 cases in 2023, with 322 confirmed cases arising from new arrival shelters, and nearly half occuring among adults 18 years and older. University of Chicago Medicine (UCM) diagnosed an average of 3.7 cases of malaria from 2010-2021, 7 cases in 2022, and 9 cases in 2023. The New Arrivals Clinical Pathway, informed by these epidemiologic changes, provides general guidance on diagnosis and treatment for high frequency infections and a basic risk assessment strategy for high consequence pathogens. It also provides instructions on how to isolate individuals based on risk factors and clinical features and directs clinicians to contact Infection Control and ID specialists when appropriate.

New Arrivals Clinical Pathway Table of Contents, Section 1: Fever in a Returning Traveler/New Arrival Schema

**Figure:**
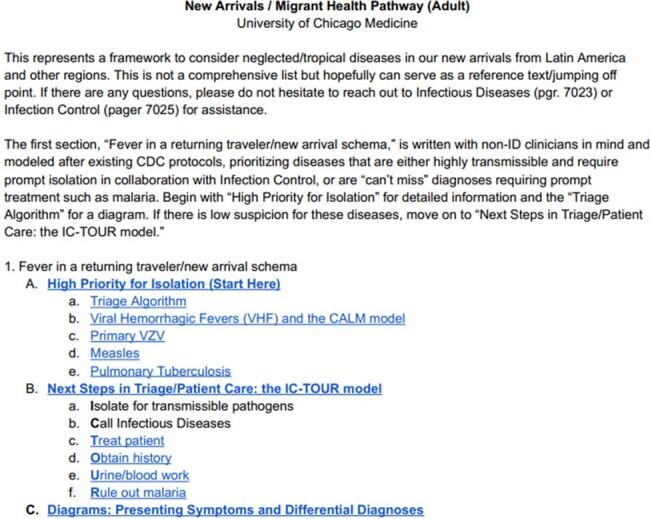

When first opening the New Arrivals Clinical Pathway, clinicians are presented with these stepwise instructions in section 1, easily navigable via hyperlinks to relevant content.

**Conclusion:**

The ongoing influx of new arrivals into Chicago, coupled with a housing crisis and congregant living conditions in shelters have rapidly changed the epidemiology of infectious diseases in Chicago, in turn creating a need for rapid education of clinicians. Using an easily accessible, readable, algorithmic model assists non-ID providers and ID trainees in triage and initial management of these increasingly common diseases.

New Arrivals Clinical Pathway Table of Contents, Section 2: List of Neglected/Tropical Diseases to Consider

**Figure:**
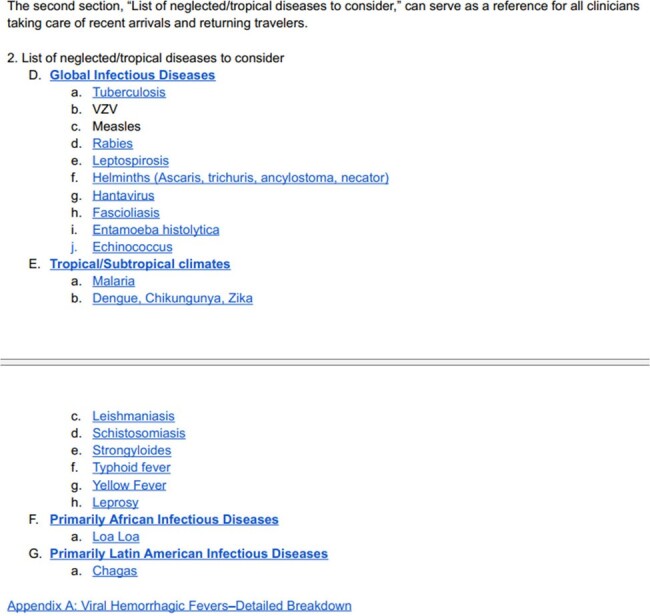

Section 2 provides a convenient reference for neglected tropical diseases for all clinicians taking care of recent arrivals and returning travelers. It is organized by geographic region, and provides details regarding diagnosis, treatment, and infection control requirements for each disease.

**Disclosures:**

**Jennifer Pisano, MD**, Beckman Coulter: Advisor/Consultant

